# Effects of UK hostile environment policies on maternity care for refugees, asylum seekers, and undocumented migrants in Camden: Examining the experiences of healthcare professionals and community organisations

**DOI:** 10.1016/j.jmh.2024.100291

**Published:** 2024-12-28

**Authors:** Poppy Pierce, Haleema Adil, Tiffany Kwok, Catherine Cooke, Deveney Bazinet, Kate Roll, Sara L Hillman

**Affiliations:** a Medical School, Faculty of Medical Sciences, University College London, London, United Kingdom; b Department of Science, Technology, Engineering and Public Policy, University College London, London, United Kingdom; c Institute for Innovation and Public Purpose, University College London, London, United Kingdom; d Institute for Women's Health, University College London, London, United Kingdom

**Keywords:** Healthcare access, Health policy, Immigration policies, Maternity care, Hostile environment, Migrant health

## Abstract

**Background:**

The London borough of Camden has long been home for many refugees, asylum seekers, and undocumented migrants (RASUs). Over time, it has witnessed an increase in the population of these migrant groups, accompanied by notable changes in the obstacles they encounter when seeking health services, particularly maternity care. We explore how the ‘hostile environment’ policies affect access to and delivery of quality maternity services for RASUs.

**Methods:**

This study was conducted over eight months (November 2021–July 2022) both remotely and face-to-face, in various locations in Camden and in the Maternity Department at University College London Hospital, UK. Healthcare professionals (HCPs) and community organisations (COs) were identified as two major stakeholders involved in the care provision for RASUs. 33 semi-structured interviews were conducted (with 22 HCPs and 11 COs) to understand their experiences of delivering care to this population.

**Results:**

There was consensus among HCPs and COs that the current immigration policies undermined their duty of care, personal morals, and the principles of the NHS. These policies have created a restrictive environment, making it increasingly difficult for migrants to navigate the healthcare system and creating an atmosphere of distrust, propagating fears of being charged. This has led to HCPs and COs going beyond their remits to ensure that RASUs are accessing and engaging with maternity care, regardless of an individual's status and despite any potential repercussions for themselves.

**Conclusion:**

In the face of an intensifying hostile environment under the UK government, supporting RASUs cannot be solely reliant on political measures. We need to advocate for healthcare navigator roles, health justice partnerships, specialist teams, and comprehensive training for service providers. HCPs and COs should be adequately supported in their endeavours to ensure RASUs have access to standardised, high-quality maternity care.

## Highlights

• Camden's growing refugee, asylum seeker, and undocumented migrant population faces increasing barriers to maternity care access.
• Immigration policies undermine NHS care principles and create distrust among migrants.
• Healthcare professionals and community organisations exceed remits to ensure migrants access adequate quality maternity care.
• Advocacy is needed for healthcare navigator roles and comprehensive training for care providers.

## 1 Introduction

Access to healthcare for refugees, asylum seekers, and undocumented migrants (RASUs) is notoriously impacted by the UK's hostile environment policies, created by then Home Secretary Theresa May in 2012 (Griffiths and Yeo, 2021). The government aimed to establish immigration policies to foster "a really hostile environment for illegal immigrants (Grierson, 2018)." These were chiefly enacted through the 2014 and 2016 Immigration Acts (McMahon, 2023), with the primary goal of limiting access to fundamental public services and opportunities, including housing, education, employment, and healthcare (Taylor, 2018).

These hostile environment policies are pervasive and their effects on maternity care are now being understood but are conflicting with national guidance. In 2016, the National Maternity Review Better Births report presented a vision for advancing maternity care with an emphasis on patient-centred care and improving maternal outcomes (Better Births, 2016). NHS trusts have also had to respond to recommendations from the MBRRACE-UK 2022 report, which highlighted that maternal mortality rates amongst black women were 3.7 times higher compared to their white counterparts (Knight et al., 2022). These reports played a role in reshaping priorities within the NHS, however national commitments to better health outcomes in maternity care will not be achieved while the hostile environment policies exist (Jones et al., 2022).

The Home Office, a ministerial department tasked with overseeing security and economic prosperity in the UK (GOVUK, 2021), is responsible for managing the majority of these policies. However, the implementation of the policies also relies on other government departments, as well as public services and communities. As one article outlined, “[the hostile environment policies] turn regular citizens and public workers into immigration officers, making them complicit in the enforcement of the hostile environment (LGSMigrants, 2022).” This has raised many ethical and clinical issues among healthcare professionals (HCPs), who have faced significant pressures to police patients’ needs for care (Essex et al., 2022). Over a brief span of time, the extensive implications of these policies have resulted in a less welcoming society with increased uncertainty for migrants. To combat this challenging landscape created by the hostile environment policies, numerous community organisations (COs) have emerged (Benwell et al., 2023). They have become integral to bridging gaps in support, providing essential services, and fostering a sense of belonging for those navigating the complexities of displacement (Martinez-Damia et al., 2023).

Numerous migrant groups exist in the UK, prompting legitimate concerns that the hostile environment policies may have created conditions in which an individual's immigration status could negatively impact their access to essential services, such as healthcare (the definitions for each status group are outlined in Table 1). RASUs are extremely vulnerable, with complex social, physical, and mental health needs (Lebano et al., 2020). These issues are exacerbated by the multitude of barriers RASUs face when accessing healthcare, with adverse pregnancy outcomes being correlated to inequalities in the provision of maternity care to RASUs (Heslehurst et al., 2018).

**Table 1:** Different migrant population groups and their entitlements to NHS healthcare.

Migrant population group	Entitlement to healthcare
Refugees: Individuals in the UK who have received refugee status, which is a five-year leave to remain (Refugee Council, 2023). Individuals with this status have either come through a UN resettlement programme, where they receive full refugee status on arrival, or they have been given this status as part of a successful asylum claim (Refugee Council, 2023).	All healthcare is free.
Asylum seekers: Individuals who have come to the UK through “irregular means,” meaning outside of laws, regulations, and international agreements, and have made an asylum claim with the Home Office (Refugee Council, 2023). Asylum seekers are those who have an active application open or those who have submitted an appeal and are awaiting the outcome. Someone who has been “refused asylum” has had their application rejected and they have yet to submit another application, or they are considered “appeals rights exhausted (Maternity Action, 2023).” There are multiple outcomes to a successful asylum claim, which include leave to remain, humanitarian protection, and other forms of leave (GOVUK, 2022).	For active asylum seekers*,* all healthcare is free. For refused asylum seekers primary care and accident and emergency care are free; secondary and tertiary care are chargeable, which includes maternity care (unless they qualify for an exemption).
Undocumented migrants: Individuals in the UK without legal status and who have not actively made themselves known to the Home Office (Liberty Human Rights, 2019). The term covers a wide range of circumstances, including those who are struggling to keep up with application fees, those who do not have the resources to challenge a Home Office decision, or those without the legal support to navigate a changing and complex set of immigration rules (Liberty Human Rights, 2019). It also includes those who were dependent on an abusive partner and leaving the relationship meant they lost their status (Pellegrino et al., 2021).	Primary care and accident and emergency are free; secondary and tertiary care are chargeable, which includes maternity care (unless they qualify for an exemption).

Table 1. Different migrant population groups and their entitlements to NHS healthcare

The RASU population has been increasing in the UK with the annual asylum applications reaching 132,000 at the end of December 2022 (Observatory, 2023). The London borough of Camden, with a diverse population exceeding 210,000 (Office for National Statistics, 2022), has continually been a welcoming place for migrant groups to settle (Camden Council, 2023), hence it is important to examine care provision here. The main hospital in Camden is University College London Hospital (UCLH), a university-affiliated tertiary centre with approximately 6000 births annually (NHS Choices, 2022). Extant literature has highlighted the prominent role of both HCPs and COs as care providers for RASUs (Mangrio and Sjögren, 2017; Refugee Council, 2024; World Health Organization, 2021), yet there is limited research that elucidates their individual experiences of delivering care within the hostile environment. This study was conducted to gain an understanding of the HCPs’ and COs’ attitudes towards the hostile environment policies and how they provide care to RASUs under these policies.

This study aimed to explore how hostile environment policies impact access to and delivery of quality maternity services for RASU populations. It sought to describe the situation from the perspectives of HCPs and COs and to offer recommendations for improving services for this group. It was felt that the specific political climate and influx of refugees into the borough of Camden at the time of this study placed additional burdens on service providers and hence provided further rationale for carrying out this work. Hence, the objective of the study was evaluated through semi-structured interviews with two of the key stakeholder groups, HCPs and COs.

## 2 Methods

This qualitative research was conducted over eight months (November 2021–July 2022) both remotely and face-to-face, in various locations within Camden and in the Maternity Department at UCLH.

Local ethical review was undertaken through internal hospital governance procedures and this work was registered and carried out ​​as a NHS service evaluation. HCPs worked within the NHS Trust and all COs were embedded partners within the UCLH Maternity Service. Procedures for obtaining informed verbal and written consent from all participants and ensuring anonymity adhered to UCL ethical standards and had been reviewed and approved by the High Risk UCL Ethics Committee. Participants were informed that their involvement was entirely voluntary.

The interview questions were designed to reveal the intricacies and nuances of how immigration status affects access to maternity services and how care is delivered to RASUs. To elicit detailed insights into the experiences and challenges faced by both HCPs and COs, interview questions covered topics such as experiences of working with RASUs; perceived barriers related to migration status; understanding of immigration policy; workplace environment and dynamics; engagement between community and healthcare sectors; training received on RASUs' care; and individual conduct in delivering care to RASUs. The dimensions covered in the interviews were selected based on a combination of factors, including a review of relevant literature, input from subject matter experts, and preliminary discussions with stakeholders involved in RASU care.

For HCP interviews, opportunistic sampling was employed by approaching HCPs working within the Maternity Department who were willing and able to take part. For CO interviews, purposive sampling was used to select participants based on several criteria: their organisation's affiliation, direct involvement with RASUs, partnership with the UCLH Maternity Service, and availability.

Information about the project was verbally explained to participants and then followed up with an email. Verbal and written consent was then obtained. Researchers then scheduled private interviews with the participants at their place of work or online via the Zoom platform. All interviews were conducted in English. Interviews were audio recorded digitally on Zoom and transcribed verbatim using Zoom transcription software. Researchers then analysed transcripts to ensure they accurately reflected the audio recording of the interviews.

The participant sample size was not predetermined. The aim was to conclude recruitment upon reaching data saturation — when no new sub-themes were determined from thematic analysis. After completing 33 interviews, notable repetition of concepts was observed in the coding, confirming that thematic saturation had been achieved. It was determined that further sampling would not provide additional insights related to the primary aim of the interviews.

The interview transcripts underwent analysis using an inductive thematic approach to identify overarching themes. Researchers independently read transcripts, created notes, and established initial codes within the Dedoose software. These codes were further refined and applied, following Braun and Clarke's 6-step framework for qualitative analysis (Clarke and Braun, 2013).

## 3 Results

**Chart 1:**
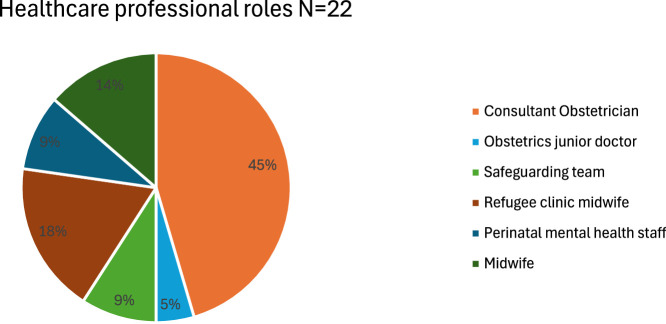
Healthcare professional roles.

**Chart 2:**
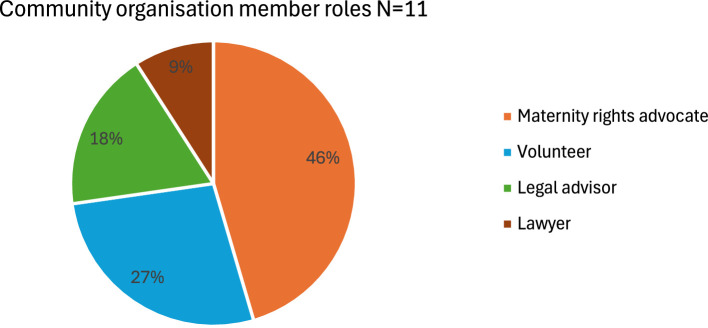
Community organisation member roles.

In total, 33 interviews were completed (Med = 50 min; range = 35–90 min). Professionals included 22 HCPs and 11 COs. All interviewees identified as female. At the time, professionals had worked in their current position for a range of 2–15 years (Med = 4 years). Each participant outlined their specific roles, as presented in Chart 1, Chart 2. HCPs described the broad spectrum of services they provided to RASUs. For example, one professional focused on perinatal mental health and employment support, while another assisted families with immigration issues and coordinated with housing teams, GPs, and schools. Several obstetric midwives specialised in safeguarding vulnerable pregnant women by connecting them with local COs for comprehensive care. COs also addressed diverse areas, including maternity rights advocacy, immigration guidance, and provided baby banks. For instance, a lawyer representative helped migrant families with asylum applications, while a refugee advocate addressed poor housing conditions and relocations. Additionally, volunteers informed women about their maternity care entitlements and ensured they received appropriate referrals.

Overall, interviews revealed three main themes in which the hostile environment policies affect access to and delivery of maternity care: (1) through specific policies which directly impact RASUs’ access to maternity care; (2) through the pressure these policies place on HCPs and COs when delivering care, and (3) how HCPs and COs have had to adapt their provision of care to circumnavigate these challenges.

In relation to (1), policies directly impacting RASUs’ access, three primary hostile environment policies were identified through responses; namely the NHS charging programme, the dispersal policy, and the information-sharing policy.

### *NHS charging policy*

In relation to the NHS charging policy, CO interviewees recounted instances where RASUs were billed despite meeting exemption criteria or being eligible for free maternity care. One CO interviewee highlighted that in dire situations “pregnant women dealing with other health conditions like diabetes were compelled to make decisions based on what care they could afford [CO4].” Several CO interviewees highlighted that the responsibility of bearing the charges burdened the pregnant women only, characterised by one CO as a "gendered approach to targeting vulnerable women [CO10].”

HCPs noted variations in maternity service booking procedures across trusts, which is also compounded by the variable involvement of the Overseas Visitor Team. One HCP explained that “in booking procedures they sometimes include questions about immigration status and in others we don't have to ask. The overseas team usually deals with that kind of stuff. Whatever [NHS] trust you're in, the process is immensely unstreamlined and lacking clarity [HCP2].”

Most interviewees had negative impressions of the Overseas Visitors Team. One HCP described them as “aggressively pursuing chargeable individuals and frequently intruding at inconvenient times to check status [HCP6]." One remarked that "the team is an entity you know exists but don't really understand [HCP11],” while another said, “they're just a group of people with a credit card machine [HCP1].” The team was criticised for making decisions without considering patient context or clinical situations. This sentiment was echoed by COs, who shared stories of patients feeling anxious about visiting the maternity ward for fear of being charged, even when they had no reason to be concerned.

While involvement with the team varied, HCPs expressed concerns of increasing interaction with the Overseas Visitors Team over the years. HCPs revealed they have recently felt increasingly burdened with the responsibility of identifying those who should be charged, despite it being unrelated to the maternity care they aim to provide. All HCPs emphasised that billing and immigration regulation are not their responsibilities. As one HCP put it, “my job is to care for women, not police their care [HCP20].” Others stressed that “charging is meant to be a separate system and immigration status shouldn't be mentioned during care [HCP14].” Many HCPs, specifically midwives, stated that they “tend to ignore issues of migrant entitlements and the overseas team [HCP9].”

In summary, the NHS charging policy has created significant challenges, adding undue stress on pregnant women and healthcare providers, complicating the provision of maternity care, and highlighting the need for clearer processes and better support for vulnerable women.

### *Dispersal policy*

Several interviewees highlighted the frequent and abrupt relocations of pregnant asylum seekers, sometimes at term, with minimal notice and insufficient information about the reasons for their relocation. One HCP noted, “pregnant refugees were moved as late as 39 weeks into their pregnancy, which was incredibly unsafe given the proximity to birth [HCP18].” Another added, “[women] wouldn't turn up for appointments, only for us to find out they had been relocated, even at over 35 weeks [HCP13].” Several interviewees shared stories of patients being relocated multiple times, forcing them to navigate different maternity care systems, some of which were more migrant-friendly than others. These relocations often disrupted continuity of care, with HCPs expressing concerns about missed appointments. One HCP mentioned that “so many of our patients attend their first antenatal appointment in the third trimester, and some avoid care altogether until it's critical [HCP22].” COs emphasised that relocation disrupts trust and community building, further stressing vulnerable women. Continuity of care was seen as vital but not always possible, with one HCP stating it “helps build and maintain trust with these vulnerable women [HCP3].” Another noted, “certain communities share knowledge about the healthcare system because someone found the service safe and recommended it. If we can make sure women have positive experiences then it really helps strengthen relationships [HCP15].”

### *Information sharing policy*

HCPs and COs had a collective concern regarding the sharing of information and surveillance within the delivery of healthcare, with COs specifically noting an increase in information sharing between the NHS and the Home Office. Numerous HCPs raised concerns regarding the appropriate management of immigration-related information should it emerge during the delivery of care. One HCP was shocked upon realising that consultations with HCPs contributed to an immigration case, information that they had assumed would be kept confidential. Additionally, it was revealed that information sharing is not always intentional. One CO interviewee pointed out that “mistakes are made when it comes to sharing information with the Home Office [CO8].” CO interviewees recounted occasions when the Home Office were investigating information and data that was not legally required for them to collect. An example discussed by both COs and HCPs was the rescinding of a 2017 policy requiring passports to be shown on first antenatal visits. Following its reversal, one HCP explained that the change was not adequately communicated, resulting in “HCPs and admin members of the team not knowing what they should be asking for when booking appointments [HCP5]”. In conclusion, HCPs and COs share significant concerns that increased information sharing risks breaching patient confidentiality and negatively impacting immigration and health outcomes.

(2) Pressures on HCPs and COs in delivering care

Both HCPs and COs frequently cited a lack of knowledge and training about relevant policies, exacerbated by poor communication and inconsistent guidance from the Home Office and NHS trusts. Many HCPs felt unprepared to implement these policies, with one stating, “I should be able to quote [the guidance], but I can't. I'd have to go in and check it and that doesn't feel great [HCP10].” COs also noted that recent policies have restricted rights and complicated their work. The hostile environment policies have created a climate of uncertainty, complicating maternity care for RASUs. One CO explained the current provision and its impact on patient care: “UK healthcare provision is often devolved and fragmented, making it a complex system infinitely more challenging to navigate, especially for professionals without training in immigration law. I've spoken to patients where the people looking after them made dangerous decisions [CO11].” This confusion among staff can lead to harmful and incorrect decisions, as many HCPs lack a full understanding of these policies.

(3) How HCPs and COs adapt care to navigate challenges

Interviewees reinforced the immense responsibility associated with looking after vulnerable individuals, often necessitating the undertaking of further tasks to make sure that “these women are not going to slip through the cracks [HCP17].” Despite their limited capacity to do so, all HCPs were driven to actively seek out services. Numerous interviewees explained that they would write letters of support for housing applications and even accompany RASUs to the housing authority, regardless of whether it would have any impact.

Some HCPs personally purchased baby clothes and essentials until charity support was arranged, while others arranged taxis for RASUs to ensure they attended appointments. However, these actions were conditional based on HCPs availability amid their clinical duties. Moreover, HCPs would “write things down for people, or draw out directions so that people know what they can access locally, like food banks or charities [HCP4]”. One HCP detailed their experience of providing care even in questionable scenarios, “sometimes I'll see pregnant women off the record when it's not strictly regarding maternity care, especially if there is any doubt about whether or not they are entitled to care. It worries me because I know it's something that I could potentially lose my licence over, but my role has [been], and always will be, to provide care [HCP16].” One HCP noted, “I wouldn't tell certain people that I do give care when I shouldn't [HCP7]” and another HCP shared, “I feel like I could get into trouble, which is silly because it is my duty to offer care [HCP19].” Overall, despite the risks to their professional licences, HCPs often choose to provide care in uncertain situations, driven by their commitment to patient well-being over any concerns for bureaucratic constraints.

Alongside the healthcare provision, the CO sphere has also responded significantly to aid in supporting RASUs. One CO interviewee explained, “as organisations, we are trying to expand our available services that we are offering to these women and families. We never used to deal with accommodation, but this is an issue that they are dealing with so we are supporting them as much as we can [CO9].” Innovative methods have also been utilised to help women overcome charging and bills, as one CO recounted, “creating a cash payment plan for an undocumented migrant, which then allowed the bill to be written off as cash to be paid at one penny per week, which is not accepted by their charging department [CO1].” Such instances have highlighted the development and flexibility of the CO role in aiding RASUs, despite it being beyond the original remits of their role. Unfortunately, with the expansion of their roles without adequate funding and guidance, one CO interviewee highlighted: “this creates undue pressure on charities and community organisations and sadly this can result in incorrect or misleading information as staff are under trained and overworked [CO6].” One HCP corroborated this, explaining “I had a patient who received incorrect entitlement advice from an organisation and so they would avoid appointments thinking they might be charged. These mistakes can actually damage the health of RASUs [HCP8].”

## 4 Discussion

The focus of this project has been on the impacts of hostile immigration policies on the experiences of HCPs and COs caring for RASUs. The empirical data highlights the pervasive effects of the policies and practices that permeate into the healthcare sphere, restricting access to maternity care for RASUs and subsequently requiring HCPs and COs to urgently fill in the gaps.

A key finding was the impact of the NHS charging policy and the role of the Overseas Visitors Team. Introduced in 2014, the NHS charging programme requires trusts to impose fees on certain groups for secondary and tertiary healthcare, including maternity care. This includes asylum seekers whose claims have been denied and undocumented migrants (Department of Health, 2014). While exemptions exist to protect vulnerable individuals (NHS Choices, 2023), proving eligibility falls on them. This study highlights that the NHS charging policy uniquely affects women simply because they can become pregnant, unlike their male counterparts. The average cost of maternity care is £7000 per person, but this can significantly increase to as much as £14,000 for complex cases (Jones et al., 2022). According to governmental data, migrant women are disproportionately charged more than men overall (GOVUK, 2023), which is predominantly due to charging for maternity care, where only mothers, not fathers, are charged (Pellegrino, 2023).

In most NHS trusts, the Overseas Visitors Team identifies individuals subject to charges (NHS England, 2023). HCPs expressed concerns that these teams, despite having limited clinical understanding, often have final discretion in decisions. Many viewed the team negatively, noting its adversarial approach to pursuing payments, which can instil fear and worsen mental health in RASUs (Pellegrino et al., 2021b). This reputation is compounded by evidence of frequent misunderstandings of regulations and failure to exempt eligible pregnant RASUs from charges (Feldman, 2017). A 2022 Doctors of the World report found that over a third of pregnant migrant women using its services had been charged for healthcare, often inappropriately (Politis, 2022), highlighting the failure of policy safeguards and the growing issue of wrongful charging (Pellegrino et al., 2021b).

Although the charging system and maternity care are separate processes, many interviewees reported involvement in determining a patient's eligibility for free healthcare. While organisations like Doctors of the World and the British Medical Association offer training on migrant entitlements (DOTW, 2022; BMA, 2023), there is no mandatory education for maternity care providers across NHS trusts. Alongside a lack of training, most HCPs and COs felt the charging programme contradicts their professional values, a concern supported by existing literature (Birthrights and Birth, 2019; BMA, 2021). This additional responsibility can detract from clinical care and undermine the patient-provider relationship, making it harder to keep RASUs engaged (Bertolini, 2021). Ethical conflicts arise, as HCPs believe in providing care under any circumstance, while legal uncertainty clouds their ability to do so (Bertolini, 2021; Onarheim et al., 2021). Both HCPs and COs indicated they would prioritise the women's well-being, even at the risk of their own jobs, a behaviour mirrored by other professionals who often "turn a blind eye" or exploit "loopholes" to exercise discretion despite institutional rules (Strabmayr et al., 2012; Miklavcic, 2011; Suphanchaimat et al., 2015).

Numerous HCPs highlighted that charging for care was inadvertently impacting the quality and delivery of care. These findings are consistent with numerous reports that have highlighted the negative effects of the NHS charging programme (Feldman, 2017; Walker and Farrington, 2021; Academy of Medical Royal Colleges, 2019), such as detering access and resulting in poorer health outcomes for parents and their children (Feldman, 2017). Maternity Action, a UK-based advocacy group for maternity rights, found that charging leads to avoidance of healthcare because of a fear of charges and the humiliation of being refused care (Roscoe, 2020). According to the MBRRACE-UK 2019 report, three women died between 2015 and 2017 who may have been reluctant to access maternity care because of fears about charging and its impact on their immigration status (Knight et al., 2019). Whilst there is limited research that quantifies the effects of charging on patient morbidity or mortality (Rassa et al., 2023), there is evidence to demonstrate that late bookings and non-attendance to appointments, due to fears of being charged, negatively impact clinical outcomes for patients (Saunders et al., 2022).

The dispersal policy was also identified as a major factor contributing to disruptions in pregnancy, violations of RASUs’ rights, and undermining of continuity of care. Asylum seekers are initially placed in hostels or hotels before being moved to temporary accommodations, often with multiple relocations during the asylum process (UK Visas and Immigration, 2016; British, 2023). The Health Access for Refugees Programme 2021 report enforces that to uphold RASU's rights, pregnant individuals should only ever be moved once if ever, not during the "protected period" of 34 weeks of pregnancy until 6 weeks postpartum, and with at least 10 days' notice (Refugee Council, 2021). While Home Office policy supports minimising disruptions during late pregnancy, these rights are frequently violated (Phillimore, 2016; McKnight et al., 2019; Maternity Action, 2013; Refugee Council, 2021), compromising continuity of care.

All interviewees emphasised the importance and effectiveness of ensuring continuity of care for RASUs, by maintaining the same midwifery team throughout their pregnancy (Bradford et al., 2022). Qualitative research focused on refugee women within the UK highlighted higher satisfaction ratings where continuity of care was maintained (Bulman and McCourt, 2002). This is crucial for RASUs as it fosters trust-building and enhances their ability to navigate the healthcare system more effectively (Royal College of Midwives, 2023; Perriman et al., 2018). This being said, the quality of interactions with HCPs significantly influences how well this continuity is received (Mangrio and Sjögren, 2017; Higginbottom et al., 2019). Positive experiences are shared within communities, encouraging engagement, while negative ones deter it. Building trusting relationships between RASUs and HCPs is essential for holistic care and sustained engagement (Ningsih et al., 2023; Barkensjö et al., 2018; Johnstone et al., 2016); however, the current dispersal policy hinders this process.

Another policy of concern is the information-sharing policy, which requires NHS trusts to report individuals with outstanding debts over £500 for more than two months without a repayment plan (Maternity Action, 2019). This can affect immigration applications and lead to the deportation of undocumented migrants, creating fear and deterring them from seeking care (Rassa et al., 2023; Asif and Kienzler, 2022). The complex and ever-changing policies have led to confusion and errors, particularly in maternity care. Although NHS Digital and the Home Office ended data sharing in 2018 (House of Commons and Social Care Committee, 2019), updates were poorly communicated to HCPs and COs. The repeal of the 2017 policy requiring passports at first antenatal visits further contributed to uncertainty, preventing RASUs from seeking care and confusing HCPs about booking requirements (Worthing et al., 2021). Overall, the complexity of information-sharing policies hinders care and risks misinterpretation, which potentially jeopardises patient safety and HCPs' roles (Canning, 2021).

Despite the challenges posed by the hostile environment, HCPs and COs have gone beyond their typical roles to prioritise quality maternity care for RASUs. The delivery of these services is multifactorial, with various issues contributing to poorer experiences for some RASUs (Asif and Kienzler, 2022). HCPs have taken a holistic approach, addressing both health-related and non-health-related challenges, which are closely tied to RASUs' engagement with maternity care (Nellums et al., 2021).

HCPs noted that limited communication pathways with COs increased the pressure to find beneficial services for pregnant RASUs. Despite their limited capacity, many HCPs actively sought out these services, a commitment that one study found to be closely tied to their level of clinical autonomy (Harrison and Daker-White, 2019). Examples from other studies show HCPs making adjustments to facilitate RASUs' needs, such as referring clients to charities or ordering tests under the doctor's name (Allen et al., 2013; Priebe et al., 2011). While navigating bureaucratic barriers is crucial for supporting RASUs, it adds to HCPs' workloads. Recognising HCPs as facilitators and advocates can improve outcomes (NHS Health Education England, 2016), but effective communication with COs is essential to reduce pressures and ensure early involvement.

Overall migration numbers are rising significantly (Migration Observatory 2024), but legal aid funding is declining (KFF, 2023; The Law Society, 2023; Turn2Us, 2023). CO interviewees noted large shifts in the community sector, which have not always been well-received. Increased demand has led to outsourcing of immigration services to non-government organisations like Migrant Help for legal support (Migrant Helpline, 2023) and private companies for asylum seeker accommodation (Bulman, 2019). Organisations such as Maternity Action have expanded their services, including legal teams for wrongful charging cases (Maternity Action, 2023). While these adjustments help manage the legal workload, they also strain resources, potentially affecting service quality and leading to incorrect or misleading information being given (Help, 2022). Despite their efforts, HCPs and COs face growing pressure and rising demand, underscoring the need for improved support and coordination.

## 5 Recommendations

Although changes to hostile environment policies are crucial to limit their impact on maternity care, recommendations have been outlined to improve support and access to maternity care for RASUs under the current provisions. Furthermore, the existing gap between the overburdened healthcare and community sectors needs to be abridged through sharing of resources and advocating for new roles to better facilitate collaborative care.

One suggestion is a healthcare navigator role within the hospital setting to support patients in accessing hospital care and COs, which aligns with NHS aspirations for healthcare provision in general, and especially for vulnerable groups (NHS Health Education England, 2016). This role is designed to encompass various responsibilities and is pivotal in assisting individuals in accessing timely and appropriate support to effectively address diverse needs.

It is also crucial to decouple healthcare access from immigration policies, which can be achieved through health-justice partnerships (UCL, 2023). Incorporating legal support into multidisciplinary teams can streamline referral pathways to the relevant COs. The tenet of these partnerships will allow HCPs to effectively respond to and prioritise individual health needs, with less administrative demand.

By introducing healthcare navigator roles and fostering collaborative health-justice partnerships in all hospital settings, the burdens on both healthcare and community sectors can be partially relieved, which would enable more patient-focused care and holistic support for vulnerable individuals (Budde et al., 2021; Beardon et al., 2021).

Within the healthcare sector, advocacy and funding are needed for specialised HCP roles or teams that can holistically care for the multifaceted needs of RASUs. These specialised teams would enable complex social needs assessments whilst maintaining continuity of care. Another facet to consider is the creation or reinforcement of training programmes that support HCPs to establish a baseline level of knowledge to boost their confidence whilst delivering more standardised care to RASU populations. This could include updates in guidance about migration pathways to the UK, information on how they can support referrals for temporary accommodations and transport services, and details on how to identify unmet legal or welfare concerns.

## 6 Strengths

The study addresses a pressing and contemporary issue in the UK, particularly in the context of the hostile environment policies affecting RASUs. By focusing on how these policies intersect with maternity care, this study provides insights into a critical area of public health that is both timely and relevant. In addition, the study hones in on a specific population—RASUs in Camden—and their unique challenges in accessing maternity services. This focused approach allowed for a deeper understanding of the specific barriers these groups face, which can inform targeted interventions. Furthermore, the use of semi-structured interviews allowed for in-depth exploration of the experiences and perspectives of HCPs and COs. This qualitative approach was well-suited to capturing the nuanced impacts of immigration policies on service delivery and personal experiences. Lastly, the study involved a broad range of participants, including 22 HCPs and 11 representatives from COs. This diversity of perspectives enhances the robustness of the findings, as it incorporates viewpoints from both clinical and community support angles.

## 7 Limitations

In interviews, HCPs and COs emphasised the significant role of the Overseas Visitors Team yet attempts to engage with the team at UCLH were unsuccessful. This unaddressed role represents a significant gap that warrants exploration in future research. It is also imperative to hear directly from RASUs to develop a detailed and nuanced understanding of their experiences. The current absence of their voices remains a limitation in this research. Incorporating RASUs’ perspectives is vital to inform maternity care tailored to their needs and foster stronger relationships with HCPs and COs. Lastly, as this study focuses on Camden and UCLH, it pertains to only one patient population, and HCPs’ and COs’ attitudes may differ across communities. Since RASUs' experiences vary tremendously, these recommendations may not universally apply, but they aim to lay a foundation for improving access to and delivery of maternity care for RASUs.

## 8 Conclusion

This study affirmed existing concerns about the unjust circumstances faced by pregnant RASUs due to the hostile environment policies. While the maternity experiences of RASUs' vary, and the challenges they face differ, HCPs and COs collectively acknowledged that these ethically fraught policies obstruct the delivery of compassionate maternity care to these populations.

Whilst the hostile environment was significantly intensified under the previous UK government, its effects remain rampant as ever and the new government is yet to combat the policies in place. Hence, supporting RASUs cannot be solely reliant on political measures. We need to advocate for healthcare navigator roles, health justice partnerships, specialist teams, and comprehensive training for service providers. Importantly, HCPs and COs should be adequately supported in their significant endeavours to ensure RASUs have access to standardised, high-quality maternity care.

## Ethics approval and consent to participate

Local ethical review was undertaken through internal hospital governance procedures and this work was registered and carried out ​​as a NHS service evaluation. Healthcare professionals worked within the NHS Trust and all community organisations were embedded partners within the UCLH Maternity Service. Procedures for obtaining informed verbal and written consent from all participants and ensuring anonymity adhered to UCL ethical standards and had been reviewed and approved by the High Risk UCL Ethics Committee. Participants were informed that their involvement was entirely voluntary.

## Consent for publication

Not applicable.

## Availability of data and materials

The datasets used and/or analysed during the current study are available from the corresponding author on reasonable request.

## CRediT authorship contribution statement

**Poppy Pierce:** Writing – review & editing, Writing – original draft, Visualization, Validation, Software, Resources, Project administration, Methodology, Investigation, Formal analysis, Data curation, Conceptualization. **Haleema Adil:** Writing – review & editing, Writing – original draft, Visualization, Validation, Software, Resources, Project administration, Methodology, Investigation, Formal analysis, Data curation, Conceptualization. **Tiffany Kwok:** Writing – review & editing, Software, Resources, Project administration, Methodology, Investigation, Formal analysis, Data curation, Conceptualization. **Catherine Cooke:** Writing – review & editing, Software, Resources, Project administration, Methodology, Investigation, Formal analysis, Data curation, Conceptualization. **Deveney Bazinet:** Writing – review & editing, Software, Resources, Project administration, Methodology, Investigation, Formal analysis, Data curation, Conceptualization. **Kate Roll:** Writing – review & editing, Supervision, Funding acquisition, Conceptualization. **Sara L Hillman:** Writing – review & editing, Supervision, Funding acquisition, Conceptualization.

## Declaration of competing interest

The authors declare that they have no known competing financial interests or personal relationships that could have appeared to influence the work reported in this paper.

## Appendix Supplementary materials

Image, application 1
