# Supplementary material for: Effects of UK hostile environment policies on maternity care for refugees, asylum seekers, and undocumented migrants in Camden: Examining the experiences of healthcare professionals and community organisations
Source: J Migr Health. 2024 Dec 28;11:100291. doi: 10.1016/j.jmh.2024.100291 (PMC11751534; doi:10.1016/j.jmh.2024.100291)
Supplement: Supplementary file 1 [file mmc1.pdf]

University College London Hospitals
NHS Foundation Trust

Women's Health Division
250 Euston Road
2nd Floor North Wing
London
NW1 2PG

Direct line: 020 3447 2521
Fax: 020 3447 9565

Website: www.uclh.nhs.uk

25th August 2024

TO WHOM IT MAY CONCERN

Regarding submission of paper to Journal of Migration and Health

"Effects of UK hostile environment policies on maternity care for refugees, asylum seekers, and undocumented migrants in Camden: examining the experiences of healthcare professionals and community organisations"

This letter is to confirm that the above work was undertaken as a service evaluation and quality improvement initiative within the maternity services department within the Division of Women's Health at UCLH NHS Foundation Trust. The work was registered with the Trust Clinical Audit and Quality Improvement Committee as one of a number of pieces of parallel work across the local maternity service relating to evaluation of service provision for vulnerable pregnant women.

Please do let us know if any other information is required.

Yours sincerely,

Melissa Whitten

Melissa Whitten

Governance Lead, Women's Health Division UCLH
Consultant in Obstetrics and Fetal Medicine UCLH
UCL MBBS Year 5 Module B & Year 3 IBSc Women's Health Lead

Women's Health Division, UCLH NHS Foundation Trust, 2nd Floor North, 250 Euston Road, London NW1 2PG

melissawhitten@nhs.net
